# Nature of Pre-Earthquake Phenomena and their Effects on Living Organisms

**DOI:** 10.3390/ani3020513

**Published:** 2013-06-06

**Authors:** Friedemann Freund, Viktor Stolc

**Affiliations:** 1 GeoCosmo Science Group, SETI Institute, Mountain View, CA 94043, USA; 2 Department of Physics, San Jose State University, San Jose, CA 95192, USA; 3 Earth Sciences Division, NASA Ames Research Center, Moffett Field, CA 94035, USA; 4 Space Biosciences Division, NASA Ames Research Center, Moffett Field, CA 94035, USA; E-Mail: viktor.stolc-1@nasa.gov

**Keywords:** pre-earthquake phenomena, positive holes, ULF (ultralow frequency) waves, ELF (extremely low frequency) waves, air ionization, water oxidation, hydrogen peroxide, physiological effects

## Abstract

**Simple Summary:**

Earthquakes are invariably preceded by a period when stresses increase deep in the Earth. Animals appear to be able to sense impending seismic events. During build-up of stress, electronic charge carriers are activated deep below, called positive holes. Positive holes have unusual properties: they can travel fast and far into and through the surrounding rocks. As they flow, they generate ultralow frequency electromagnetic waves. When they arrive at the Earth surface, they can ionize the air. When they flow into water, they oxidize it to hydrogen peroxides. All these physical and chemical processes can have noticeable effects on animals.

**Abstract:**

Earthquakes occur when tectonic stresses build up deep in the Earth before catastrophic rupture. During the build-up of stress, processes that occur in the crustal rocks lead to the activation of highly mobile electronic charge carriers. These charge carriers are able to flow out of the stressed rock volume into surrounding rocks. Such outflow constitutes an electric current, which generates electromagnetic (EM) signals. If the outflow occurs in bursts, it will lead to short EM pulses. If the outflow is continuous, the currents may fluctuate, generating EM emissions over a wide frequency range. Only ultralow and extremely low frequency (ULF/ELF) waves travel through rock and can reach the Earth surface. The outflowing charge carriers are (i) positively charged and (ii) highly oxidizing. When they arrive at the Earth surface from below, they build up microscopic electric fields, strong enough to field-ionize air molecules. As a result, the air above the epicentral region of an impending major earthquake often becomes laden with positive airborne ions. Medical research has long shown that positive airborne ions cause changes in stress hormone levels in animals and humans. In addition to the ULF/ELF emissions, positive airborne ions can cause unusual reactions among animals. When the charge carriers flow into water, they oxidize water to hydrogen peroxide. This, plus oxidation of organic compounds, can cause behavioral changes among aquatic animals.

## 1. Introduction

Earthquakes are caused by the build-up of tectonic stresses. When rocks reach a critical limit, catastrophic failure occurs, causing a rupture to propagate, sometimes over distances of hundreds of kilometers, with typical speeds in the range of 2.2–3 km/s—supersonic with respect to the speed of sound in air. The rupture generates shockwaves in the Earth that spread at speeds of about 6 km/s for the compressional (P) waves and about 3.4 km/s for the more destructive shear (S) waves. An earthquake of magnitude 7 releases an energy equivalent to that of more than 2000 Hiroshima-class atomic bombs. The magnitude scale being exponential, the energy released by a magnitude 9 earthquake is equivalent to more than 2,000,000 A-bombs. 

Mainstream seismology, as currently practiced, focuses on understanding earthquakes from a mechanistic viewpoint using information collected by the analysis of past earthquakes to predict future earthquakes [1,2,3]. Unfortunately, even if the database for past events is very large, counting thousands, even tens of thousands, the prediction of any single future earthquake is subject to the large uncertainty, which is an inescapable corollary of statistics of small numbers. For this reason seismological prediction models are typically given with uncertainty windows on the order of decades, even centuries. Only in very few cases have predictions been attempted with quoted uncertainty windows of only a few years [3,4]. Seismologists are of course aware of these statistical limitations. This is why their majority believes that earthquakes are inherently unpredictable [5,6]. 

In view of the enormously large amounts of mechanical energy released by major seismic events, however, it is inconceivable—from a physics perspective—that the Earth would not produce some identifiable non-seismic and non-mechanical signals prior to such large-scale catastrophic events. Whether or not, and to what extent, the precursory signals are reliable is another question.

There have been countless accounts of precursory signals, too many to list here. They span a bewildering diversity of phenomena ranging from stories of unusual animal behavior on land, in the air and in the water, to the emission of electromagnetic radiation over many orders of frequency space, to distinct changes in the electron distribution at the lower edge of the ionosphere some 100–300 km above the surface of the Earth, and many others. 

The problem facing earthquake forecasting is not that there has been a lack of reported pre-earthquake (pre-EQ) phenomena. The real problem lies in the lack of understanding what the different types of reported pre-EQ phenomena mean and how they might be generated. 

There have also been countless attempts to interpret pre-EQ signals and to assign them to known physical processes that could occur in the Earth crust in response to the build-up of tectonic stresses. The problem is that the reported pre-EQ phenomena are so diverse and fall into the competency range of so many different scientific disciplines that it has not been possible, so far, to reach any consensus.

If such a situation arises, namely that scientists from different disciplines cannot agree on a scientific explanation of a set of naturally occurring phenomena such as pre-EQ phenomena, chances are that the physical underpinning of the processes in the Earth crust during the build-up of tectonic stresses are not yet properly understood. This is particularly relevant to the contentious issue of unusual pre-EQ animal behavior reported in the scientific and non-scientific literature. 

### 1.1. Setting the Stage

Earthquakes are usually reported in terms of the location of their epicenter, their magnitude, and their depth. The hypocenter is defined as the point at depth, where the rupture starts. The epicenter is the point on the Earth surface straight above the hypocenter. 

Most major crustal earthquakes occur in the 7–35 km depth range. At this depth the isostatic overload is substantial, ranging from about 2,000 kg/cm^2^ to 10,000 kg/cm^2^, 2–10 metric tons on every square centimeter. Under these conditions it is highly questionable whether two processes, which are widely quoted in the context of pre-EQ signals, can in fact take place: (i) microfracturing and (ii) the electrokinetic effect. 

Microfracturing had been introduced in the late 1960s as part of the “Dilatancy theory” [7,8], which started with the observation that, when rock cylinders are uniaxially stressed, they bulge out and that their total volume increases [9,10,11,12]. In unconstrained rock cylinders such volume increase is due to internal cracks opening as a result of the applied stress. However, deep in the Earth crust, under the isostatic load of tens of kilometers of rock, the opening of cracks or microfractures is unlikely because the attendant volume increase has to perform work against a very large overload.

Rocks in the upper reaches of the Earth crust, from zero to about 5–7 km, have non-zero porosity. The pores are water-filled or brine-filled. They often communicate, allowing the fluids to flow along stress gradients. Such fluid flow leads to separation of charges because cations tend to be retained on the walls of pores and narrow channels, while anions are carried along with the flow. This is the electrokinetic effect. The resulting potential, the “streaming potential”, produces voltage signals, which have been invoked to explain geoelectric and electromagnetic signals recorded prior to major earthquakes [13,14,15,16]. However, streaming potentials can only be produced in the most shallow crust, where rocks are porous but where earthquakes do not typically occur. 

Nonetheless microfracturing and the electrokinetic effect have been invoked in the context of unusual pre-EQ animal behavior. 

A third process frequently quoted in the context of pre-EQ phenomena is emanation of radon gas from the ground. Radioactive ^222^Rn, which has a half-life of 2.8 days and decays via emission of a 507 MeV alpha particle into polonium ^218^Po, is a product of the uranium-radium decay chain. Changes in pre-EQ radon emanation have long been reported [17,18,19]. Each alpha particle emitted during radon decay creates about 10^5^ electron-ion pairs along its path, forming a narrow plasma channel, about 1 cm long in air. A small fraction of these electron-ion pairs escape recombination and, hence, lead to an increase in air conductivity. This air conductivity increase has been claimed to be responsible for a number of pre-EQ phenomena including animal behavior [20]. The idea is that, if changes in radon emanation are due to microfracturing, animals might possess unusual capabilities to detect the faint vibrations from microfracturing in the near-surface environment. 

Much of what has been said and written about pre-EQ phenomena can be traced back to the seismology and its reliance on mechanical concepts. 

Microfracturing, for instance, is based on the assumption that pre-EQ stresses can be transmitted laterally from the hypocenter over long distances defined as “earthquake preparation zone” where the radius r is given by the relation r = 10^0.43M^, M the magnitude and r the distance in kilometers [21]. For a magnitude 7 earthquake r would be 1,000 km. Such large EQ preparation zones and the assumed lateral stress transfer are inconsistent with other reports that pre-EQ stress changes are seldom detected, even with most sensitive strain gauges [22,23,24,25,26].

The electrokinetic effect is likewise based on mechanical concepts, namely that stress gradients provide the driving force for fluid flow through porous rocks, which in turn would generate electric signals. Fluctuations in the flow rate are then expected to lead to low frequency electromagnetic emissions [14]. 

Many other attempts to find explanations for reported pre-EQ phenomena also rely on mechanical concepts inherited from seismology.

### 1.2. Shifting Paradigm

Largely unnoticed by the mainstream geoscience community, work over the past 3½ decades has led to crucial insight into non-seismic pre-EQ signals. The original goal of this work was to characterize inconspicuous defects in minerals: pairs of oxygen anions that have changed their valence from the common oxidation state 2- to the more oxidized state 1-. These so-called peroxy defects occur in the matrix of silicate minerals in igneous and high-grade metamorphic rocks—rocks that form the bulk of the Earth’s crust over the seismogenic depth range [27,28]. 

Examples of peroxy defects are O_3_Si-OO-SiO_3_ bonds replacing Si-O-Si bonds in mineral structures. The peroxy bonds are introduced through the dissolution of small amounts of “water” into minerals that crystallize from magma or recrystallize in deep high-temperature environments. The H_2_O molecules that enter into the solid state turn into “impurity” hydroxyls, O_3_Si-OH:

O_3_Si-O-SiO_3_ + H_2_O ⬄ O_3_Si-OH HO-SiO_3_ (1)

Upon cooling, when diffusional processes freeze and thermodynamic equilibrium can no longer be maintained, the O_3_Si-OH pairs undergo a redox conversion:

O_3_Si-OH HO-SiO_3_ ⬄ O_3_Si-OO-SiO_3_ + H_2_ (2)

This is a classical redox conversion, unusual only in the sense that the electron transfer involves hydroxyl oxygens donating an electron to their protons, reducing them to H. Two H combine to H_2_, while the oxygens, now oxidized to the valence state 1-, combine to form peroxy. 

The O^−^–O^−^ bond in O_3_Si-OO-SiO_3_ is very short, only 1.5 Å, about half the distance between normal neighboring O^2−^ anions. The shortness makes the peroxy bonds susceptible to the perturbations such as the slightest sliding of mineral grains during the build-up of stress, causing O_3_Si-OO-SiO_3_ bonds to break. The broken peroxy bonds split off electronic charge carriers, chemically O^−^ in a matrix of O^2−^, physically defect electrons in the O^2−^ sublattice, known as positive holes and symbolized by h^•^.

The h^•^ are highly mobile and have the capability to flow out of the stressed rock volume deep in the Earth crust, where a rock rupture may soon occur. The h^•^ flow out into the surrounding rocks. They travel fast and far, tens of kilometers, possibly hundreds of kilometers prior to large earthquakes. They form electric currents in the Earth crust, sometimes hundreds of thousands of amperes strong [29]. When these currents fluctuate, they emit electromagnetic (EM) radiation. The ultralow frequency (ULF) components of these EM waves can be transmitted through tens of kilometers of rocks and detected at the Earth surface. Likewise the attenuation of ULF and ELF waves through ocean water is weak. Hence, the effects discussed here would also apply to cases where earthquakes occur offshore. 

When h^•^ charge carriers arrive at the Earth surface or cross from the solid Earth into water, they cause a variety of reactions, physical and chemical. How these reactions express themselves in the surface environment is of interest in the context of animal response and the topic of this report. 

## 2. Pre-Earthquake Processes and the Biosphere

Reports on unusual animal behavior before major earthquakes are innumerable, some dating back to antiquity. Because so little is known about the nature of the physical or chemical clues to which animals appear to respond, there is a strong tendency in some scientific circles to dismiss the reports and call them “anecdotal”. However, if similar observations, often strikingly similar, have been reported across centuries by people living on different continents, in different seismically active regions, a reasonable argument can be made that these reports are not just “anecdotal”. Since they are validated by multiple independent observations, they suggest the presence of physical and/or chemical stimuli in the environment, to which animals respond. For moderate to large earthquakes the Earth appears to produce these stimuli within a time window of about two weeks to one day, sometimes only a few hours, before the event. For magnitude 9-class earthquakes the time window could be wider. 

### 2.1. The Effect of Electromagnetic Waves on Organismal Physiology

Exposure to electromagnetic waves can have physiological effects on a wide variety of organisms [30]. Importantly, the physiological effects can be induced by exposure to the level of EM energy in the thermal noise, which helps to explain the challenge in analytical detection of earthquake precursors in the EM spectrum. For example, one of the most astonishing effects of extremely low amplitude EM oscillating field, even when the magnetic flux of such field is below the thermal noise (*i.e.*, nanoTesla field), is to change the electron precession, or spin, in universally conserved biochemical reactions that coordinate genome expression to maintain circadian timing. The cryptochrome proteins, which maintain the circadian rhythm, also show high sequence similarity to universally conserved proteins that maintain genome integrity by repair of oxidation-damaged DNA [31]. Such weak EM fields tuned in frequency to the rate of electron spin (*i.e.*, MHz) have been shown to cause aberrant growth of cells and disorientation with respect to the magnetic and gravity vectors in various organisms, including plants and animals [32]. Therefore, the universal time-setting basis of invariant biochemical reactions can be an EM field in the thermal noise.

Additionally, there have been many studies over several decades reporting on significant physical, biological and health-related effects associated with changes in solar and geomagnetic activity. The changes in solar and geomagnetic activity lead to changes in the ambient extremely low frequency (ELF) and the ultralow frequency (ULF) field at the Earth surface, specifically to the immersion of the Earth surface environment into broadband EM field (0.1–50 Hz), which can be the causal link between these changes in the ambient ELF field, both under pre-earthquake conditions and during geomagnetic storms [33], and the various effects noted in the biosphere.

However, despite decades of research in characterizing the effects of non-ionizing EM radiation on organismal physiology, including in humans, the results often seem to be inconsistent or difficult to interpret. For example, some experimental studies claim a direct effect of ELF on metabolism [30], while theoretical studies dispute the possibility of such effects based on first principles [34]. Additionally, the international guidelines for limiting exposure to time-varying electric and magnetic fields in the low-frequency range consider ULF and ELF EM fields to be biologically relevant only, if their amplitudes exceed the ambient field of about 40 pT by at least 10^9^ times (*i.e.*, in excess of 0.01 T magnetic flux density) [35]. How can this discrepancy in empirical evidence be reconciled without dismissing the potential importance of modulating organismal physiology with EM fields, while remaining in accord with physical principles? 

It is important to recognize that EM waves are time-dependent phenomena and their effects on temporally organized biochemical cycles in living cells, such as the reduction oxidation cycle, can generate quite opposite effects depending upon their phase relationships. The redox cycle, also called the metabolic cycle, is the fundamental biochemical process that controls the timing of all biochemical reactions in living cells, including energy production, RNA transcription, and DNA replication. Through temporal coordination a chemical conflict between the reductive and oxidative reactions is avoided, while at the same time a coherent or interfering transfer of energy is enabled between the ambient natural electromagnetic (EM) field and living cells. The interaction between EM waves and electron transport in redox reactions clearly depends on the phase relationship between oscillating systems. 

Therefore, whether or not ambient non-ionizing EM fields are able to affect biological processes in organisms can be very sensitive to resonance, which occurs when the organismal physiology is able to transfer energy from ambient EM field in tune with the electromotive force across membranes. An appropriate experimental approach to reconcile the inconsistencies in the effects of EM field on organismal physiology is to identify the point of resonance between the time-setting basis of ambient EM waves in the environment and the phase of the biochemical reduction oxidation cycle in the electron transport chain (ETC) *in vivo*. 

Clearly, animals including birds and turtles can detect the Earth’s magnetic field, and use the field to navigate during migration [36]. For example, the Bar-tailed Godwit bird has one of the longest non-stop migrations of any species, up to 10,200 km (6,300 mi). Magnetic perception can also account for the behavior of cows and wild deer that tend to align their bodies north-south direction while relaxing, but not when the animals are under high voltage power lines that generate ELF electromagnetic fields, which disrupt the magnetic alignment of ruminants [37,38]. Although the geomagnetic field is weak and is generally thought to be masked by thermal fluctuations and other noise, the proposed bio-physical mechanism for magneto perception is based on coherent quantum effects of extremely weak magnetic fields on the electron spin in each of radical pair intermediates of molecules in redox reactions [32]. Depending on the electron spin state, different reaction products are produced, and at different rates. The switch between the products of the redox reactions is shifted by the ambient EM field, which can affect the organismal behavior [32]. 

These results indicate that the rates and direction of electron transfer reactions—the rate at which an electron can move or jump from one chemical species (called the electron donor) to another (called the electron acceptor)—are subject to ambient EM field influences. This process of electron transport *in vivo* is fundamental to all organisms because it is biochemically coupled with the transfer of H^+^ ions (protons) across a membrane. The resulting electrochemical proton gradient is used to generate chemical energy in the form of adenosine triphosphate (*ATP*). Importantly, the universal ETC is made from components (such as iron oxides, e.g., heme) with a strong magnetic moment that increases the effect of the weak ambient EM field [32]. 

Intriguingly, the rotation frequency of the molecular enzyme motor called ATP synthase, which synthesizes ATP also oscillates at ELF (10–70 Hz) [39]. Consequently, thermodynamic coupling by EM resonance is possible between the ambient EM field and the most fundamental biochemical process of life. Although the energy and geometric scale of external EM waves and the energy and dimensions of the ATP synthase enzyme motor differ by at least 12 orders of magnitude, it is to be noted that, due to quantum mechanical delocalization of electronic wave functions, the ATP synthase has the capability to extract EM energy from a volume much larger than the volume, which itself occupies [40,41]. This astonishing physical phenomenon allows the systems to exceed—through athermal mechanisms—the limit on EM radiation with intensity less than 10 mW/cm^2^ thought to be unlikely to significantly affect physiology. In addition, it is important to note that pausing the rotation of ATP synthase, or changing the direction of its rotation, leads to the production of reactive oxygen species (ROS) and to the depletion of ATP, respectively. Both biochemical events result in metabolic stress *in vivo* (e.g., oxidative stress) that can conceivably account for anomalous animal behavior.

Therefore, organisms are likely able to detect earthquake-induced variations in the ambient EM field because EM perturbations can affect the time-setting physical basis for the known *universal* biochemical order in living cells [42]. 

### 2.2. Surface/Subsurface Charges, Air Ionization, and Corona Discharges

Positive holes are positive charge carriers. As h^•^ travel through the rocks, they repel each other and “try to get away from each other” as far as possible. The surface of the Earth is as the furthest they can go. The h^•^ become trapped at the surface, forming a subsurface charge layer of thickness d, where d is typically only a few ten nanometers. This charge layer is characterized by a surface potential V, typically on the order of 2–3 V [43]. 

As rocks become ever more stressed by the tectonic forces deep below, the more h^•^ charge carriers are generated. The more h^•^ charge carriers arrive at the surface, the more they compress the subsurface charge layer so the d decreases. 

The electric field E is defined as potential V divided by the thickness d, E = V/d. If V is, say, 2 V and the thickness of the h^•^ subsurface charge layer is 40 nm (4 × 10^−7^ cm), the E field at the surface will be on the order of 5,000,000 V/cm. As laboratory experiments demonstrate, air molecules, presumably O_2_, can become field-ionized under these conditions, meaning that they become so highly polarized in contact with the ground that they lose an electron to the surface:

O^−^|_surface_ + O_2_|_air_ => O^2−^|_surface_ + O_2_^+^|_air_ (3)

When this process sets in, the near-surface air becomes laden with positive airborne ions.

It has been shown experimentally that, when a block of rock is mechanically stressed at one end, the air at the other end becomes indeed positively ionized [43]. The number of positive ions can reach values on the order of 10^6^ cm^−2^ s^−1^. This is much higher than the “fair weather” ion content in normal air [44]. Field data recorded by air ionization sensors collocated with the ULF search coil magnetometers of the QuakeFinder stations along the San Andreas Fault system in California and along the subduction zone in southern Peru have confirmed massive, positive, pre-EQ ionization of air and coincident EM pulses [45,46]. 

It is important to note that the ions generated by the mechanism described by Equation (3) are all positive. Positive airborne ions have been known for a long time to have a strong physiological effect on animals and humans [47,48]. Therefore it should not come as a surprise that animals in the ground, on the ground and in the air would respond to high positive airborne ion concentrations in their environment. 

When stresses continue to increase, the voltages associated with the surface/subsurface charge layer increase further and d is further reduced. If the voltage V reaches, say, 3 V, and the thickness becomes 6 nm, the E field will reach values on the order of 50,000,000 V/cm. This is enough to accelerate electrons in the air, which are always present due to cosmic ray and other ionization events, to such high values that they begin to impact-ionize neutral air molecules, triggering tiny corona discharges. Importantly these corona discharges produce avalanches of electrons and positive ions in the air. The free electrons will attach themselves rapidly to neutral air molecules such as N_2_, forming negative airborne ions such as N_2_^–^. In addition the corona discharges produce tiny light flashes with a broad emission spectrum over the VIS and UV regions [43]. The UV component of these flashes can be expected to produce ozone, O_3_, and other reactive gases such as nitrous oxides, to which animals may respond [49]. 

### 2.3. Oxidizing Water to Hydrogen Peroxide at the Rock-Water Interface

From a physics perspective h^•^ are charge carriers, *i.e.*, defect electrons on the oxygen anion sublattice, but from a chemistry perspective they are O^–^, equivalent to •O radicals and highly oxidizing. When h^•^ cross the rock-to-water interface, they oxidize H_2_O to H_2_O_2_:

O^−^|_surface_ + H_2_O|_liquid_ => OH^−^|_surface_ + •OH|_liquid_ (4)

Two •OH combine to form H_2_O_2_. The reaction is stoichiometric [50]. 

The formation of H_2_O_2_ at the rock-water interface, presumably also at mud-water interfaces, due to the arrival of h^•^ charge carriers flowing down a stress gradient prior to a not-too-distant earthquake can be expected to have some effect on animals living in the water. 

During a multi-year study of the spring mating behavior of the European toad *Bufo bufo* in the San Ruffino Lake, central Italy, about 75 km from L’Aquila, it was noted that the toads disappeared from their mating and spawning ground starting about 5 days before the magnitude 6.3 L’Aquila earthquake of April 6, 2009, and did not return until a series of aftershocks had subsided [51]. While it has been suggested that animals may have acquired, through evolution, the ability to take evasive action before potentially deadly earthquakes [52], in the case of the toads in Lake San Ruffino near L’Aquila their pre-EQ departure from the aquatic environment was more likely triggered by chemical clues in the water such as the release of H_2_O_2_ and possibly other oxidation products resulting from an influx of h^•^ [51]. 

Humboldt squid stranding are not rare events. In late 2012 several massive strandings of mostly juvenile Humboldt squids occurred on the shores of Monterey Bay in Northern California [53]. In this case the strandings appeared to coincide with red algae blooms. Certain red algae can produce the potent neurotoxin domoic acid, a derivative of the cyclic amino acid proline, which deregulates the Ca balance in nerve cells [54]. Domoic acid accumulates in the marine food chain. It can be harmful, even lethal, to sea mammals and sea birds, and it can also affect squids, which feed on fish that have fed on domoic acid-producing red algae [55]. 

There have been reports on squid strandings ostensibly related to earthquakes. One particular case involved a modest magnitude 4.0 earthquake that struck July 14, 2009 at 7:34 am local time about 35 km offshore near La Jolla, San Diego, CA at a depth of about 20 km. Many large mature Humboldt squids washed up on the shore on the same day, dazed and dying [56]. William Gilly, Stanford University Hopkins Marine Station, reported that strandings of the same species had been seen along the same stretch of the Pacific coast during the last three days prior to the earthquake. Humboldt squids typically live at 100 m water depth and deeper. If a red algae bloom is an unlikely cause, the question arises whether something might have happened at the ground-water interface, which displaced these squids.

One possibility would be that, during the influx of h^•^ into the water column at the ground-water interface, oxidation reactions occur in addition to the simple H_2_O_2_ production as described by Equation (4). These additional reactions could lead to enhanced degradation of organic compounds in the sea bottom mud or in the near-ground water [57] possibly converting benign compounds to neurotoxins such as domoic acid or to other compounds, which might be toxic to specific groups of animals. Further work will be needed to address the complexity of the electrochemically driven oxidation reactions at the ground-to-water interface caused by the influx of stress-activated h^•^ charge carriers from below. 

### 2.4. Oxidation Reactions in the Soil

#### 2.4.1. Ozone

There have been many reports on major gas and trace gas emanation related to earthquake activity. Most commonly there is an increase in the efflux of CO_2_, or in the efflux of CO_2_, H_2_, N_2_, He, Ar, CH_4_, often with isotopic signatures suggesting an upper mantle origin [58]. In the context of pre-EQ animal responses these gases can probably be disregarded, but some other trace gases may be more relevant such as ozone, O_3_. 

**Figure 1:**
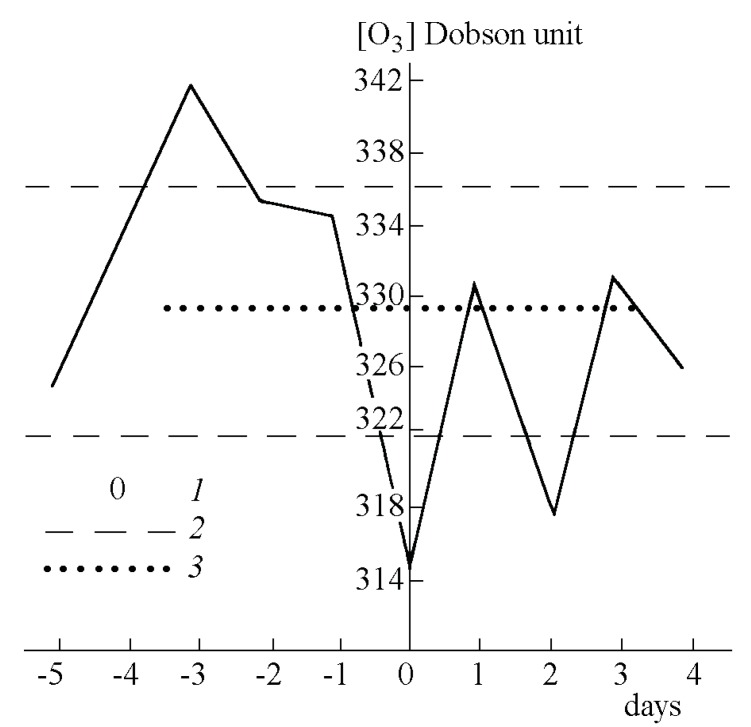
Ozone level changes associated with 88 strong earthquakes of magnitudes 4.5–7.2 in Central Asia from 1973 to 1985 (solid line) [59]. A Dobson unit is the column density of O_3_ in Earth atmosphere equivalent to a 10 µm thick layer of O_3_ under standard temperature and pressure. Dotted line: average O_3_ values during non-perturbed days; Dashed lines: 95% confidence level.

A rise in the ozone levels during or after large earthquakes has been reported and interpreted as due to cracking of rocks at or near the surface with attendant formation of ozone through fracture-induced electric discharges [49]. However, there is also a report for a noticeable rise in ozone levels days before major earthquakes, when surface or near near-surface fracturing of rocks could not have occurred. Figure 1 shows the average of regional ozone levels 5 days before to 4 days after 88 strong earthquakes of magnitudes 4.5 to 7.2 that occurred in Central Asia from 1973 to 1985 [59]. The data were collected at fixed ground stations often hundreds of km from the epicenters. The measured O_3_ concentrations, given in Dobson units, represent variations that must have been observable over large regions. On the average, for the 88 reported events, elevated O_3_ contents are observed 2–4 days before with a maximum 3 days before these earthquakes. 

By plotting the O_3_ concentration in Dobson units Figure 1 gives the total O_3_ content throughout the atmosphere. Most of it, of course, refers to the O_3_ layer. Its variability is indicated by the two dashed lines for the 95% confidence level. The “high” 3 days before the earthquakes and the “low” on the day of the earthquakes represent local sources, presumably near the ground level.

The laboratory experiments mentioned above had indicated that, when we stressed rocks up to failure, we observed first exclusively positive airborne ions and second a mixture of positive and negative airborne ions in about equal numbers. This second step arose from corona discharges at the edges and corners of the rock samples [43]. 

If we apply these findings to situations in the field we expect that, prior to large earthquakes, massive amounts of positive airborne ions would be emitted from the Earth surface around the epicentral region, followed by tiny corona discharges spreading over an equally wide region. Though the light emitted by those tiny corona discharges will probably be too weak to be noticed by the naked eye during daytime, these corona discharges can be expected to lead to ozone formation. The data reproduced in Figure 1 are consistent with this second step. In addition, co-seismic emissions of light flashes have been recorded in real-time during a recent nighttime major earthquake in Peru [60].

Animals exposed to both steps, to massive positive air ionization and ozone production at ground level, can be expected to exhibit abnormal behavior in response to the appearance of these irritants in their environment at higher levels than normal. In the case of positive air ions the response of animals could be triggered by their effect on the seratonin level in the blood, a stress hormone, [48]. Ozone, a trace gas with an acrid smell even at concentration levels as low as 40 nmol/mol or 40 ppb, is recognized by the US Environmental Protection Agency as an inflammatory agent through induction of oxidative stress [61]. 

#### 2.4.2. Radon

Another trace gas is radon, which emanates from the ground due to the radioactive decay of radium, an element in the uranium decay series and found in relative highest concentrations in granites and similar igneous rocks, which form the bulk of the Earth crust. The radium isotope ^226^Ra decays by alpha particle emission to ^222^Ra, which itself is radioactive, decaying through the emission of 507 MeV alpha particles to the polonium isotope ^218^Po [62]. ^222^Ra has a half-life of 2.8 days. 

Radon is a noble gas and widely thought to be chemically inert, released from the ground only because, prior to major earthquakes, microfracturing is supposed to occur in the near-surface underground. This is the usual mechanical concept inherited from seismology, where cracks are believed to open in the rocks, releasing gases. However, because radon is the heaviest of the noble gases, it must be chemically reactive, even more so than xenon, forming compounds with soil organics. Therefore, on its diffusion path from below to the surface through the overlying soil, radon atoms are expected to be retained due to chemisorption to soil organics. The most likely retention sites would be triple-bonded carbon compounds in the soil, forming R-Rn-C≡C-H (with R being -CH_3_ *etc.*) similar to the stable Xe analog H-Xe-C≡C-H [63]. If this is the right concept, the arrival of h^•^ charge carriers in the soil layer over regions of impending earthquake activity will preferentially attack the triple carbon bonds, oxidize them to double or single bonds, and thereby release the radon [64]. The radon atoms would then be free to continue their diffusional path to the surface, where they enter the air space.

It is true that, besides cosmic rays, radon decay in the near-surface air is the main source of the ambient air ionization accounting for about 200 ion pairs per cm^3^ air at sea level [65]. Modest changes in the rate at which radon escapes from the ground have been widely reported in relation to pre-EQ activity [17,18,19,20]. However, while radon in the near-surface air surely has an impact on all living organisms through radiation damage to their genes, there is little or no evidence that changes in the rate of radon release and the ensuing changes in the ion concentration in the near-surface air can be sensed by animals and could have an effect on pre-EQ animal behavior. 

#### 2.4.3. Carbon Monoxide

There are other forms of oxidation reactions, which a pre-EQ influx of h^•^ charge carriers into the soil layer can cause and which could incite an animal response. There is, for instance, a well-documented case where carbon monoxide (CO) was massively released from the ground prior to the magnitude 7.6 Gujurat earthquake of January 26, 2001 in North-West India. 

Using data collected by the MOPITT (Measurements of Pollution in the Troposphere) sensor onboard the NASA Earth Observing System (EOS) Terra satellite, Singh *et al.* [66] retrieved the CO concentrations in the atmospheric column over a 3-month period that included the time of the Gujurat earthquake. The Terra satellite passes over the region twice a day at 705 km altitude, once during daytime and once at night. It can provide CO concentration values with a horizontal resolution of 22 km and a precision of about 10%. CO profiles can be obtained in the absence of substantial cloud cover. 

**Figure 2:**
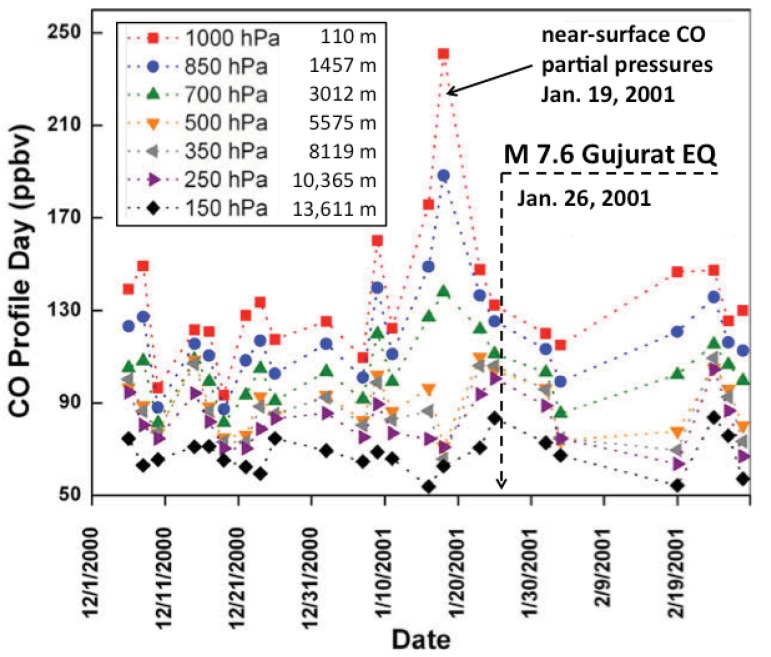
CO concentration values representing averages for a 1° × 1° area (approximately 100 × 100 km^2^) around the epicenter of the Gujurat earthquake of January 26, 2001. The CO profile is broken down into the 7 altitude windows indicated in the insert.

The Gujurat results are shown in Figure 2. The CO concentration values represent averages for a 1° × 1° area (approximately 100 × 100 km^2^). The CO profile is broken down into 7 altitude windows as indicated in the insert, from 0 up to 110 m, up to about 1,500 m, up to about 3000 m, and higher. The data show that the regional background CO concentration was always highest close to the ground, *i.e.*, in the lowest altitude window, with values between 100 and 150 ppb. However, starting probably more than a week before the magnitude 7.6 seismic event, the CO concentration increased, reaching a maximum on January 19, 2001 around 240 ppbv in the air below 110 m. The total amount of CO in the air column would be enough to reach 500 ppm near the surface, a potentially lethal concentration because CO induces phase advancement into the oxidative respiratory phase of the metabolic cycle in all aerobic organisms. Indeed, CO acts in a manner similar to cyanide by binding to the heme in cytochrome c oxidase and inhibits electron transport and ATP production, which leads to an increased ROS production. This fundamental and universal mechanism of CO-induced ROS production during the redox cycle applies to all aerobic cells [67]. Therefore, a pre-EQ influx of h^•^ charge carriers into the soil layer can cause gaseous CO release, which functions as a cellular signaling molecule that attenuates metabolic cycling toward stress (*i.e.*, oxidative damage in living cells).

The increased CO emanation from the ground indeed suggests a chemical reaction involving stress-activated h^•^ charge carriers, flooding the soil layer in the epicentral region from below. Being highly mobile, highly reactive and highly oxidizing these h^•^ can be expected to “burn” organic matter in the soil. Taking -CH_2_- as representative of organic matter, we can write an oxidation reaction as:

2 O^–^|_soil_ + -CH_2_-|_soil_ => 2 H_2_O|_soil_ + CO|_gas_ (5)

This CO gas should have the isotopic ^12^C/^13^C signature characteristic of soil organic matter and not of any carbon source from deep below [68]. At the same tune it is likely that other trace gases besides CO would be formed by the same oxidation process, possibly formaldehyde or acetone, but no data appears to be available. In addition CO_2_ might be produced as the end product of the interaction of h^•^ with soil organic matter. However, given the high background of CO_2_, any extra CO_2_ release will be near-impossible to detect.

If CO is produced by a reaction of the type described by Equation (5), the animals most affected will be ground-dwelling animals [69]. They might be poisoned and killed by the increase in CO partial pressure to concentration levels on the order of 500 ppm, in particular inside their burrows. No reports of animals that died before the Gujurat earthquake appeared to have been published. However, there are anecdotal reports of mass dying of rats and mice in connection with a series of medium earthquakes in Northern Italy in May–June 2012. Reportedly the dead animals showed no sign of physical injury [70].

## 3. Results and Discussion

The International Commission on Earthquake Forecasting for Civil Protection (ICEF) had been convened in the aftermath of the magnitude 6.3 L’Aquila earthquake of April 6, 2009. In its Final Report [71], the ICEF commented on the question of animal behavior as follows: *“An ever-popular subject of investigation is anomalous animal behavior observed before earthquakes* [72]*. In some cases, purported precursory behaviors have been discounted by systematic studies* [73]*. Animals, including humans, do respond to signals that they can feel, such as small earthquakes that might be foreshocks* [74]*. Anecdotal evidence suggests that some animal species may have evolved “early warning” systems that allow individuals to respond in the few seconds prior to the onset of strong shaking, most probably triggered by the weak shaking of the first-arriving P wave* [75]*. Geobiologists have also speculated on the evolution of sensory systems that could detect more subtle precursory signals* [52]*. However, there is no credible scientific evidence that animals display behaviors indicative of earthquake-related environmental disturbances that are unobservable by the physical and chemical sensor systems available to earthquake scientists.”*

By opening this passage with the words “*ever-popular subject of investigation*”, used here in an obviously denigrating tone, the ICEF Final Report does not contribute in a constructive way to the discussion of issues related to animals and earthquakes. Statements like this, however, are understandable, if one takes into consideration that the majority of the ICEF members are seismologists who tend to look at pre-EQ animal responses from the mechanical perspective of mainstream seismology. This prevailing attitude makes it difficult, if not impossible, to recognize the complexity of the interactions between animals and the physical world. The real problem is not the absence of observationally well-documented facts about unusual animal behavior but a lack of understanding how the reported non-seismic pre-EQ phenomena are generated in the natural environments, and what are the non-seismic stimuli to which animals appear to respond. 

The discovery of positive hole charge carriers, h^•^, has led to insights into the physical processes that occur deep in the Earth crust during the build-up of high levels of stress and into the range of unusual properties that seem to be characteristic of these previously unrecognized electronic charge carriers in rocks [28]. 

The purpose of our paper is to draw attention to the fact that, during build-up of stresses—but before catastrophic rupture—an activation of h^•^ charge carriers takes place in rocks that form the deeper layers of the Earth crust. This is a non-seismic process that takes place during every episode of waxing stress in the Earth crust regardless of whether or not the rocks will rupture catastrophically. Stress-activation of h^•^ charge carriers, a quintessential pre-EQ phenomenon, is essential to all non-seismic pre-EQ phenomena mentioned in this paper.

As highly mobile electronic charge carriers, the h^•^ have the ability to spread out of the stressed rock volume into the surrounding less stressed or unstressed rocks. They can travel fast and far. During their propagation through the Earth crust they can generate ULF and ELF electromagnetic radiation. Upon arrival at the ground-to-air interface they can cause air ionization and corona discharges. Upon arrival at the ground-to-water interface they can act as highly oxidizing •O radicals and oxidize H_2_O to H_2_O_2_. Through coupling to the electron transport chain *in vivo*, by modulating the electron transport along the ETC, ULF and ELF waves can transfer energy, which exceeds the buffering capacity against free radicals and thereby cause oxidative stress in living organisms [76]. It was shown that, consistently, some ubiquitous environmental factors feedback directly on heredity through the ETC in organisms by leaking electrons that result in oxidative damage and genome sequence variation (*i.e.*, non-random mutation) between generations [67]. 

Animals can clearly respond to a range of different physical and chemical stimuli arising directly or indirectly from the stress-activation of h^•^ charge carriers. Indeed, understanding the nature of these enigmatic the electronic charge carriers in rocks provides insight, which was previously not unavailable, into the modulation of redox states in the Earth’s ecosystems from the global scale down to the atomic level.

## 4. Conclusions

A previously unknown or overlooked physical process takes place in rocks that are subjected to large stresses deep below in the Earth crust before major earthquakes. This process leads to the activation of electronic charge carriers, which can spread out of the stressed rock volume into the surrounding rocks, traveling fast and far. They generate electromagnetic waves, of which the ultralow and extremely low frequency (ULF/ELF) portion can be transmitted through rocks. The ULF and ELF waves have the capacity to interact with living organisms and to interfere with fundamental biochemical reactions at the cellular level. When the charge carriers arrive at the ground-to-air interface, they are able to ionize the air producing—under certain conditions—exclusively positive airborne ions. Such positive airborne ions are known to have pronounced and generally detrimental physiological effects. When these charge carriers arrive at the water-to-air interface, they oxidize the water and possibly organic compounds dissolved in the water, producing potentially hazardous toxins. Understanding the complex sequence of events taking place in the Earth crust during the lead-up to major earthquakes provides insight into the causes of unusual pre-earthquake animal behavior. 
